# Dipyridamole, chloroquine, montelukast sodium, candesartan, oxytetracycline, and atazanavir are not SARS-CoV-2 main protease inhibitors

**DOI:** 10.1073/pnas.2024420118

**Published:** 2021-02-10

**Authors:** Chunlong Ma, Jun Wang

**Affiliations:** ^a^Department of Pharmacology and Toxicology, College of Pharmacy, The University of Arizona, Tucson, AZ 85721

Li et al. (1) recently report the discovery of 16 Food and Drug Administration–approved drugs as severe acute respiratory syndrome coronavirus 2 (SARS-CoV-2) main protease (M^pro^) inhibitors. They were identified from a computational virtual screening approach using the M^pro^ as the drug target, and their enzymatic inhibition against SARS-CoV-2 M^pro^ was validated in the fluorescence resonance energy transfer (FRET)-based enzymatic assay (inhibitory constant K_i_ = 0.04 to 3.27 µM).

**Fig. 1.**
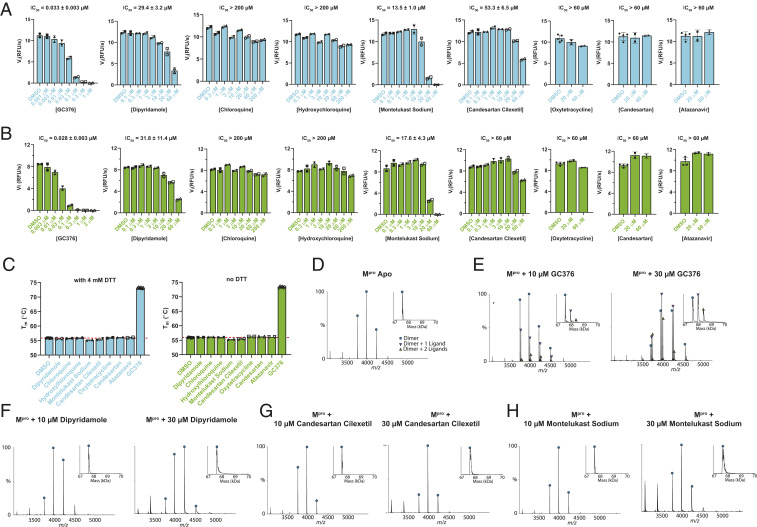
Hit validation/invalidation of dipyridamole, chloroquine, hydroxychloroquine, montelukast sodium, candesartan, candesartan cilexetil, oxytetracycline, and atazanavir as SARS-CoV-2 M^pro^ inhibitors. The most active 8 compounds out 16 identified in ref. 1 were evaluated in a FRET-based enzymatic assay in the presence (*A*) or absence (*B*) of 4 mM DTT, thermal shift binding assay (C), and native MS binding assay (*D*–*H*). The FRET-based enzymatic assay was carried out with 100 nM SARS-CoV-2 M^pro^ protein with 10 µM FRET substrate Dabcyl-KTSAVLQ/ SGFRKME-Edans (2, 4–6), the thermal shift binding assay was carried out with 3 µM M^pro^ protein and 40 µM testing compounds (2, 4–6), and the native MS binding assay was carried out with 4 µM M^pro^ protein and 10 to 30 µM testing compounds (2, 4, 5).

Among the list of 16 discovered hits, disulfiram was recently proved by us as a nonspecific promiscuous cysteine protease inhibitor that not only inhibits SARS-CoV-2 M^pro^ but also five other cysteine proteases, and the inhibition was only observed in the absence of reducing reagent, 1,4-dithiothreitol (DTT) (2). As these claimed hits do not share structural similarities with existing M^pro^ inhibitors (3), we therefore chose the eight most potent compounds, including dipyridamole, candesartan cilexetil, hydroxychloroquine, chloroquine, montelukast sodium, atazanavir, candesartan, and oxytetracycline, for the hit validation. GC376 was included as a positive control (4, 5). To rule out false positives, we tested all eight compounds in the FRET-based enzymatic assay with and without DTT, thermal shift binding assay, and native mass spectrometry (MS) binding assay. Collectively, our results have shown that, first, the most potent compound claimed by Li et al. (1), dipyridamole (K_i_ = 0.04 µM, half-maximum inhibitory concentration [IC_50_] = 0.6 µM), is a weak inhibitor of SARS-CoV-2 M^pro^ with an IC_50_ value of 29.4 ± 3.2 µM (Fig. 1*A*). However, dipyridamole did not show binding to M^pro^ in either the thermal shift assay (Fig. 1*C*) or the native MS assay (Fig. 1*F*), suggesting dipyridamole is not a potent inhibitor of M^pro^. Second, chloroquine and hydroxychloroquine did not shown inhibition against M^pro^ in the enzymatic assay either with or without DTT (IC_50_ >200 µM) (Fig. 1 *A* and *B*). They also did not show binding to M^pro^ in the thermal shift binding assay (Fig. 1*C*). Third, montelukast sodium inhibited M^pro^ with an IC_50_ value of 13.5 ± 1.0 µM in the presence of DTT (Fig. 1*A*). However, it did not show binding to M^pro^ in either the thermal shift assay (Fig. 1*C*) or native MS assay (Fig. 1*H*). This suggests that the apparent enzymatic inhibition might be a false positive. Fourth, candesartan cilexetil, candesartan, oxytetracycline, and atazanavir did not inhibit M^pro^ (IC_50_ >50 µM) (Fig. 1 *A* and *B*), nor did they bind to M^pro^ as shown by the thermal shift binding assay (Fig. 1*C*). Candesartan cilexetil also did not show binding in the native MS assay (Fig. 1*G*).

Overall, our data suggest that there might be a significant flaw with the enzymatic assay inhibition results presented in this study. None of the identified hits was confirmed to inhibit or bind to SARS-Co-2 M^pro^. A GST-tagged M^pro^ was used in their enzymatic assay; however, it is known that M^pro^ requires a native N terminus to form the enzymatic active dimer (4). DTT was not added in the enzymatic assay, but the nonspecific reactivity did not explain the results presented in the paper, as we did not observe significant enzymatic inhibition even in the absence of DTT for six of the tested compounds (Fig. 1*B*).
